# Electronic Tools to Bridge the Language Gap in Health Care for People Who Have Migrated: Systematic Review

**DOI:** 10.2196/25131

**Published:** 2021-05-06

**Authors:** Frédérique Thonon, Swati Perrot, Abhijna Vithal Yergolkar, Olivia Rousset-Torrente, James W Griffith, Olivier Chassany, Martin Duracinsky

**Affiliations:** 1 Patient-Reported Outcomes Unit (PROQOL), UMR 1123, Université de Paris, INSERM, F-75004 Paris France; 2 Unité de Recherche Clinique en Economie de la Santé (URC-ECO), AP-HP, Hôpital Hôtel-Dieu, F-75004 Paris France; 3 Faculty of Pharmacy Ramaiah University of Applied Sciences Karnataka India; 4 Department of Medical Social Sciences Feinberg School of Medicine Northwestern University Chicago, IL United States; 5 Service de Médecine Interne et d'Immunologie Clinique, Hôpital Bicêtre, F94270 Le Kremlin Bicêtre France

**Keywords:** eHealth, systematic review, migrants, health literacy, access to care, health promotion

## Abstract

**Background:**

People who have migrated or with a language barrier may face significant hurdles in accessing health care. Some apps have been specifically developed to facilitate the dialogue between health care professionals and people who have migrated who have low-level language proficiency or to promote health among people who have migrated.

**Objective:**

We conducted a systematic review to investigate development, acceptability, and effectiveness of these types of apps.

**Methods:**

We conducted a search of PubMed, Scopus, and Embase databases. We included all study designs (qualitative, quantitative, mixed) reporting development, evaluation of efficacy, or acceptability of apps facilitating dialogue with a health professional or promoting health for people who have migrated, minorities, or tourists with a language barrier, using any outcome. Two researchers selected the studies independently. We collected general information about the app, information about health literacy and cultural adaptation, information about the development of the app, evidence on acceptability or efficacy, and information on app use. Data were collected by 2 researchers independently and results were reviewed to verify agreement and reported according to PRISMA (Preferred Reporting Items for Systematic Review and Meta-analysis).

**Results:**

Positive results for translation apps included better communication, but with possible limitations, and reduced consultation time. Positive results for health promotion apps included improved quality of life and better management of chronic illnesses.

**Conclusions:**

Overall, the apps had good levels of acceptability, though only half had their efficacy evaluated. In those evaluations, the endpoints were mostly related to reported behavior change and knowledge improvement, which is common for evaluations of health promotion programs. In the future, as more health apps are created, it is essential that apps that claim to have a public health objective undergo a rigorous evaluation of their acceptability, efficacy, and actual use. Indicators of outcomes beyond changes in behavior and knowledge should be reported; change in health status or access to care should also be reported. This systematic review has helped us note the characteristics associated with improved acceptability and efficacy, which can be helpful for the development of future apps.

## Introduction

People who have migrated may face significant delays and barriers in accessing health care, especially those who do not fluently speak the language of the host country. Research has been conducted to investigate language barriers in accessing care and prevention among people who have migrated and its health consequences. Pregnant women who have migrated and with low proficiency in the language of their new country of residence have found it more difficult to access care [1], had less knowledge about the benefits of folic acid and had lower folic acid intake [2], and had a higher risk of obstetric trauma [3]. A study [4] showed that a language barrier is significantly associated with a higher occurrence of serious medical events in pediatrics. Cancer studies show less screening for colorectal cancer [5,6], cervical cancer [6], and breast cancer [6,7] among people who have migrated who had low English proficiency. Others have found that adding an interpreter to a consultation where the patient has a language barrier (any type of interpretation) results in the reduction of obstetric interventions [8], better clinical outcomes in people with diabetes [7,8], a higher rate of breast cancer and colorectal cancer screening [7], and a higher rate of influenza vaccination [7]. Overall, it has also been shown that patients with a low proficiency in the language of the country of residence receive more preventive advice and more prescriptions and have fewer emergency visits if they use interpretation services [9].

Health care professionals commonly use several solutions to communicate with people who have migrated with low language proficiency, including printed guides and brochures, informal interpreters, professional interpreters in person, professional interpreters on phone or video conference, and general translation apps. Some apps or electronic tools have been specifically developed to be used in medical consultations to facilitate dialogue between health care professionals and people who have migrated who have low language proficiency or to promote health among people who have migrated. In the last decade, there has been a sharp increase in the number of medical apps developed [10], but their impact on patients or public health should be established.

We conducted a systematic review to examine the evidence related to the development, adaptation, acceptability, and effectiveness of electronic tools designed to help health care providers communicate with or promote health among people who have migrated and who have low levels of proficiency in the language of their country or low levels of health literacy. The aim of this review was to describe the existing tools and gather evidence about features that increase the acceptability and efficacy of such tools. Our work is part of a larger project designed to develop and evaluate an app to facilitate communication between people who have migrated who face a language barrier and health professionals regarding testing for HIV and different forms of viral hepatitis [11]. Lessons learned and evidence from similar apps could help design an app that has a significant impact on public health.

## Methods

### Search Strategy, Databases, and Keywords

We wrote a protocol prior to starting the systematic review and reported the results according to the PRISMA reporting guideline [12] (Multimedia Appendix 1). We conducted a search of 3 databases of scientific publications: PubMed, Scopus, and Embase. The keywords varied according to the database used. To determine keywords, we undertook a broad preliminary search and selected some articles (between 5 and 10) identified as meeting inclusion criteria determined by the authors. After selecting these articles, we searched for their related keywords, MeSH terms (Medical Subject Headings), terms in titles or abstracts, and EmTree terms. Then the selection of keywords was tested and different combinations were tested so that the total number of results was manageable and yielded relevant articles. The final selection of keywords was critically reviewed by a university librarian. The keywords for each database are detailed in Multimedia Appendix 2.

### Inclusion and Exclusion Criteria

We used the inclusion and exclusion criteria detailed in Table 1. Although the primary focus of this review was people who have migrated, the preliminary search yielded articles reporting apps that helped bridge the language barrier for other populations, such as tourists needing emergency care and not speaking the language of the country visited or indigenous people whose primary language is different from the official language. Since these apps help health care providers communicate with patients with a language barrier, we decided to include them because we found they were relevant.

**Table 1:** Inclusion and exclusion criteria.

Criteria type				Inclusion criteria		Exclusion criteria
**Publication**						
	Language			Written in English or French		Other languages
	Date range			Published after 1998		Published before 1998
	Type			Original article, review, protocol, conference abstract, book chapter		Other types of publications: editorial, letter, notes, etc
**Study**						
	**Design**			Studies reporting the development of an electronic tool, including qualitative or quantitative studies of people who have migrated or health providers, mixed methods, literature reviews Studies evaluating the acceptability of electronic tools, including qualitative or quantitative studies, usability studies, randomized or nonrandomized trials Studies evaluating the efficacy of electronic tools, including randomized or nonrandomized trials, qualitative or quantitative studies, economic evaluations		Articles lacking information about the development or evaluation of an electronic tool Studies exploring only the perceptions of users (people who have migrated or health professionals) related to e-health or a health issue
	**Population**					
		Language	International people who have migrated not fluent with the language of the country they reside		People with no language barrier (internal people who have migrated, ethnic minorities, people who have migrated with no language barrier)	
		Communication barriers	Cultural minorities having a language barrier (eg, indigenous people whose first language is different from the official language) Tourists		People with other type of communication barriers: deaf or hard-hearing people, people with a learning disability	
	**Technology**					
			Website, mobile (smartphone or tablet) apps, other electronic technology that allows interaction with user text message or email-based services		Tools using only print material, audio, or video	
		Intervention	Technology designed to help communication between health care providers (eg, doctors, nurses, midwifes) and people who have migrated in any health care setting (eg, hospital, primary care) Technology designed to promote healthy behavior among people who have migrated		Technology that aims to facilitate communication or translation in general settings but not designed specifically for the medical setting (eg, Google Translate, apps for tourists).	
	**Outcome**					
		Development of an electronic tool	Themes emerging from interviews or focus groups, results from participants consultations		None	
		Acceptability	Comments from participants,, satisfaction surveys, data in app use or consultations		None	
		Efficacy	Changes in health outcomes (self-reported or measured with biomedical measures), changes in knowledge, attitudes, practices, and beliefs		None	

### Study Selection

A list of articles retrieved from all 3 journal databases was compiled. After excluding duplicates, 2 researchers (FT and SP) independently reviewed the title and abstracts of all documents for preselection. Articles were then reviewed in full for inclusion or exclusion; if an article was excluded, the reason was documented. We managed the selection of articles with Rayyan [13]. Differences of opinion regarding the inclusion of an article were managed by a third researcher (ORT). We also subsequently included relevant articles that were cited by articles that had been initially selected.

### Data Collection

We used 2 data collection coding sheets (Google Forms): one for each article (type of publication, year of publication, and journal) and one for each app studied (general information about the app or electronic tool, information about health literacy and cultural adaptation, information about the development of the app or electronic tool, evidence about the acceptability or efficacy of the app or electronic tool, information about the use of the app or electronic tool). All articles were read by FT, and to improve the validity of results, we performed data triangulation by having a second author read articles independently.

When articles presented additional sources of information regarding an app, such as gray literature or a website, we retrieved data about the app from this source and noted the references of this additional source of information but did not use them in the article selection.

### Quality and Risk of Bias Assessment

We evaluated the articles using ICROMS (Integrated Quality Criteria for Review of Multiple Study Designs) [14], which can be used for public health reviews that include several study designs, such as randomized controlled trials, controlled before-after, controlled interrupted times series, cluster randomized controlled trial, noncontrolled before-after, cohort studies and qualitative studies. This tool consists of 33 indicators grouped in 7 dimensions: (1) clear aims and justification; (2) managing bias in sampling or between groups, (3) managing bias in outcome measurement and blinding, (4) managing bias in follow-ups, (5) managing bias in other study aspects; (6) analytical rigor; and (7) managing bias in reporting or ethical considerations. Each indicator receives a score of 2 if the criteria for the indicator are met, 0 if this is not the case, or 1 if it is unknown whether the criteria were met. Where specified, we also found that it was necessary to use CHEERS (Consolidated Health Economic Evaluation Reporting Standards [15]), noting if the information required was available, incomplete, or not available using the same scoring system as ICROMS. It should be noted that this scoring system for protocols or medicoeconomic studies is not validated, nor is it part of the ICROMS tool.

Many articles related to eHealth are information technology usability studies. Usability studies are studies that aim to explore usability requirements, discover usability problems, and design solutions [16]. To our knowledge, there is no published article proposing a tool to evaluate the quality or bias of such studies. Usability studies mainly use either qualitative or quantitative methodologies [16], however, due to the usually small sample of participants [17] and approaches used, such as participants being asked to perform tasks and give oral feedback [18], they are closer to qualitative studies than they are to quantitative studies. Therefore, we assessed the quality and risk of bias of most of usability studies using the ICROMS tool adapted to qualitative studies, except usability studies using quantitative-only methodology, which were assessed as noncontrolled before-after. We also used the ICROMS tool to evaluate research protocols; we simply disregarded the questions that were not applicable.

## Results

### Selection

**Figure 1:**
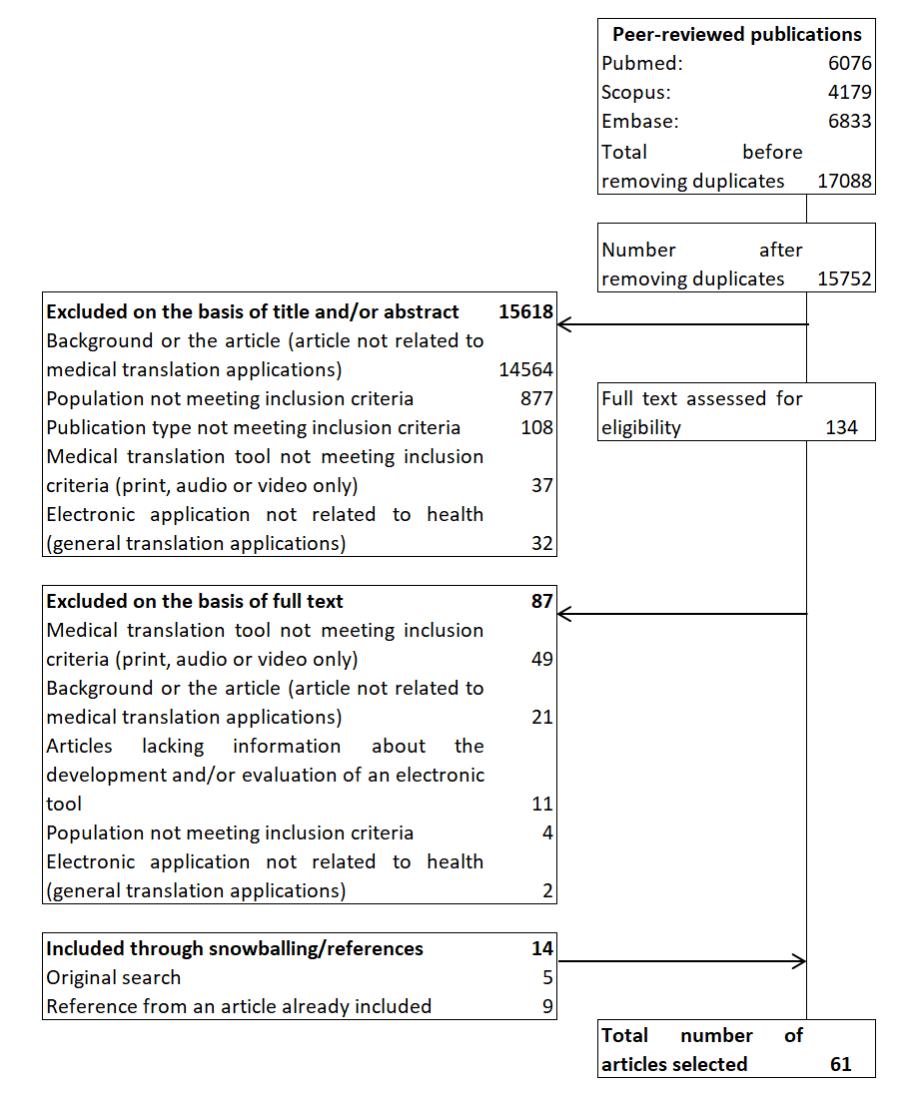
Flowchart of article selection.

The database search was carried out in October 2019. We retrieved a total of 15,752 articles from 3 databases after removing duplicates, of which 15,618 were excluded on the basis of title or abstract content. We assessed the full texts of 134 articles and excluded 87, most because the electronic tool described did not meet the inclusion criteria. We subsequently added 14 articles, because they were found either in the original background search or cited by articles that had been initially selected. In total, we included 61 articles (Figure 1). The difference between the very high number of articles originally retrieved and the number of articles selected can be explained by the fact that the original search yielded a number of articles related to translational research. The 61 selected articles were all read and analyzed by FT, and 51 (84%) were read and analyzed by a second reviewer (35 by SP and 16 by AVY).

### Characteristics

The majority of articles were original research articles (55/61, 90%); 4 were published research protocols, and 2 were proceedings of scientific conferences. Of the 61 articles, most articles (27/61, 44%) were published in journals that specialized in medical information technology, 14 (23%) were published in journals related to a disease or medical specialty, 11 (18%) were published in journals that specialized in health promotion, 6 (10%) were published in general health journals, and 3 (5%) were published in nonmedical journals that specialized in information technology. All articles were published in English. The countries of affiliation of the first authors were USA (n=40), Germany (n=5), Australia (n=5), New Zealand (n=2), United Kingdom (n=2), Italy (n=1), Nigeria (n=1), China (n=1), Switzerland (n=1), Japan (n=1), Norway (n=1), and Spain (n=1).

Of the 61 articles, 21 articles reported qualitative studies (34%), 18 articles reported usability studies (30%), 9 articles reported nonrandomized interventional studies (15%), 7 articles reported randomized controlled trials (11%), 4 articles were protocols for either randomized controlled trials (n=3, 5%) or a mixed methods study (n=1, 2%), and 2 articles reported other types of studies (1 medicoeconomic study; 1 case study).

### Quality and Risk of Bias

One article was a health economic study, for which CHEERS was used. We could not evaluate the quality of one article [19], as it is was a description of the tool and a single case study of its use on a patient. This article was included in our review because of its practical description of the tool.

The full results are available in Multimedia Appendix 3. Among the 7 randomized controlled trial articles, the mean score was 22 and the median score was 24 (the score ranges from 0 to 30, and the minimum score required for a study to be considered of robust quality is 22); 1 article did not meet the criterion to be considered of robust quality. Among the 21 qualitative studies, the average and median score were 18 and 18, respectively (the score ranges from 0 to 26, and the minimum required for a study to be considered of robust quality is 16); 7 articles did not meet the minimum to be considered of robust quality. Among the 9 noncontrolled before-after studies, the mean score was 22 and the median score was 23 (the score ranges from 0 to 30 and the minimum required for a study to be considered of robust quality is 22); 4 studies did not meet the minimum to be considered of robust quality. Among the 18 usability studies, 6 did not meet the minimum required for either qualitative or noncontrolled before-after studies to be considered of robust quality.

For the randomized controlled trials, the lowest scores were found on questions regarding allocation blinding and measurement blinding, and to a lower extent, reliability of primary outcome measures (lack of objectivity of outcome variables). For the qualitative studies and usability studies, the lowest scores were found for items of critically assessing researcher bias and lack of agreement. For noncontrolled before-after studies, the lowest scores were found regarding justification for and attempts to mitigate the lack of control group and, to a lower extent, lack of objectivity of outcome measures.

### General Characteristics of Apps

In the 61 articles, a total of 48 apps were presented (Table 2). Approximately two-thirds of the electronic tools (n=30, 63%) were developed in the USA. Other countries represented were Germany (4 apps), Australia (4 apps); New Zealand (2 apps) and Italy, United Kingdom, Nigeria, China, Switzerland, Japan, Norway, and Spain (1 app each).

Of the 48 apps, 20 apps (42%) were designed for health promotion or prevention, rather than for one specific health care setting: 11 were designed for hospital care, 8 were designed for primary care, 5 were designed for therapeutic or patient education, 3 were designed for both primary care and hospital care, and one was designed for both health promotion and primary care; 14 apps (29%) were health promotion apps that were not related to one specific medical specialty or condition, and the most represented medical specialties were cancer (9/48, 19%), mental health (6/48, 13%). Health promotion is defined as the process of enabling people to increase control over and to improve their health [20].

One-third of apps (16/48, 33%) were designed solely to facilitate interactions between people who have migrated and health care providers during a consultation, while the remaining two-thirds (32/48, 67%) were designed to promote health among people who have migrated who face a language barrier. Of those 32 apps designed to promote health, 9 were adaptations for people who have migrated of existing apps, while 23 were new apps developed specifically for people who have migrated.

Most electronic tools (38/48, 79%) were in the form of a mobile app, while other types (text messaging, website) were less common. It should be noted that 2 electronic tools (2/48, 4%) included both a mobile app and a text messaging service. Almost two-thirds of apps (31/48, 65%) were interactive, meaning that they allowed feedback from the user, either to another user or to the app.

More than three-quarters (38/48, 79%) were specifically targeted to a group of people who have migrated, and mostly for people who have migrated from specific nationalities (24/38, 63%). Over two-thirds (33/48; 69%) had 1 language in addition to the source language, nine apps had 3 to 9 languages, five had 10 to 19 languages, and one app had over 20 languages. Information about the funding of the app was available for 33 (69%) out of the 48 apps: 30 apps (63%) had received a funding from either a public source, charitable source or crowd-funding, while 2 apps (4%) had received a funding from a mix of private and public or charitable source, and 1 app (2%) had received a private or industry funding.

**Table 2:** Characteristics of apps included in the analysis.

Characteristic			Apps (n=48), n (%)
**Country of development**			
	USA	30 (63)	
	Germany	4 (8)	
	Australia	4 (8)	
	New Zealand	2 (4)	
	Italy	1 (2)	
	United Kingdom	1 (2)	
	Nigeria	1 (2)	
	China	1 (2)	
	Switzerland	1 (2)	
	Japan	1 (2)	
	Norway	1 (2)	
	Spain	1 (2)	
**Setting**			
	Health promotion/prevention	20 (42)	
	Hospital care	11 (23)	
	Primary care	8 (17)	
	Therapeutic or patient education	5 (10)	
	Primary care and hospital care	3 (6)	
	Health promotion/prevention and primary care	1 (2)	
**Medical specialty**			
	Health promotion without a focus on a medical specialty	14 (29)	
	Cancer	9 (19)	
	Mental health, psychiatry	6 (13)	
	Infectious diseases	5 (10)	
	Cardiovascular diseases, endocrinology (diabetes)	3 (6)	
	Gynecology, pregnancy	3 (6)	
	Emergency medicine, intensive care	2 (4)	
	Addiction medicine	2 (4)	
	Paramedical specialties (physiotherapy, occupational therapy, speech pathology, dietetic, podiatrists)	2 (4)	
	Pediatrics	1 (2)	
	Pulmonology	1 (2)	
**Aim**			
	Facilitating communication between migrant and health provider	16 (33)	
	Promoting healthy behavior among people who have migrated^a^	32 (67)	
	Including adaptation of existing apps	9	
**Features**			
	Mobile app	38 (79)	
	Text-messaging service	6 (13)	
	Mobile app and text-messaging service	2 (4)	
	Website for consultation	2 (4)	
**Interactivity**			
	Interactive	31 (65)	
	Not interactive	17 (35)	
**Target population**			
	People who have migrated from specific nationalities	24 (50)	
	Asylum seekers/refugees	4 (8)	
	People who have migrated at risk from their occupation	3 (6)	
	Indigenous people	3 (6)	
	People who have migrated who are concerned by a specific health condition	3 (6)	
	Other	1 (2)	
	The app does not specifically target a group of people who have migrated	10 (21)	
**Languages (in addition to the source language)**			
	1	33 (69)	
	2-9	9 (19)	
	10-19	5 (10)	
	≥20	1 (2)	
**Fee required**			
	Yes	5 (10)	
	No	9 (19)	
	Don't know	34 (71)	
**Institution funding the development**			
	Public/government	11 (23)	
	Charitable or crowdfunding	3 (6)	
	Mix of charitable and public/government	2 (4)	
	Public/government, private and charitable	1 (2)	
	Private/industry only	15 (31)	
	No information about the funding of the app	16 (33)	

^a^Of the 32, 9 were adaptations of existing apps.

### Health Literacy and Cultural Adaptation

There was information about how the translation was performed for only half of the apps (n=24); translation was performed by a professional translator (16/48, 33%); informally (4/48, 8%), usually by nonprofessional native speakers; or by a mix of professional and personal translation (4/48, 8%). Some form of quality control of the app was mentioned for 18 apps (38%), usually forward-backward translation or translation being checked by a native speaker. The translation included cultural adaptation on 23 apps (48%), half of the apps included pictures or pictograms (n=24). Two-thirds of apps had either an audio or video feature (32/48, 67%).

### Information About the Development of the App or Electronic Tool

Of the 48 apps, 33 (69%) reported using scientific methods to develop the content of the app: qualitative studies (interviews or focus groups) for 15 (45%), use of a theoretical framework such as behavioral theories for 8 (24%), use of existing guidelines of curriculums (n=5, 15%), a mix of qualitative and quantitative methods for 4 (12%), and a mix of survey and use of theoretical framework for 1 (3%).

Of the 48 apps, less than half (22/48, 46%) reported involving users (people who have migrated) in the development, 18 apps (38%) reported involving health professionals in the development, and 5 apps (10%) reported involving other stakeholders such as charities.

### Evidence About the Acceptability and Efficacy of the App or Electronic Tool

Many apps (32/48, 67%) had their acceptability evaluated (translation apps: 14/16, 88%; health promotion apps: 18/32, 56%). Of the 14 translation apps that had their acceptability evaluated, 3 were evaluated among people who had migrated only, 6 were evaluated among people who had migrated and health professionals, and 5 were evaluated among health professionals alone. Of the 18 apps designed to promote health among people who had migrated that have been evaluated for acceptability, 14 were evaluated among people who had migrated alone, 3 were evaluated among people who had migrated and health professionals, and 1 was evaluated among health professionals alone. Acceptability studies used mixed methods (20/32, 63%), used quantitative methods only (8/32, 25%), used qualitative methods only (7/32, 22%), were pilots (5/32, 16%), and randomized controlled trials (2/32, 6%). The endpoints used to measure acceptability were comments from participants (12/32, 38%), results from satisfaction survey (9/32, 28%), length of time of a consultation and satisfaction survey (4/32, 13%), data on the use of the app (3/32, 9%), a mix of survey and qualitative comments (2/32, 6%), and other outcomes (2/32, 6%). The Systems Usability Scale was mentioned in 5 evaluations. Other evaluation systems mentioned were the Technology Acceptance Model and the Stanford Communication with Physicians Scale. Among the 32 apps that had their acceptability evaluated, 25 (78%) reported an overall good or very good acceptability; 1 (3%) reported an adequate acceptability; for 3 apps (9%), the study was ongoing; and for 3 others, the results cannot be reported as they consisted of comments from participants or choice of a design. Of the 25 apps that reported a good or very acceptability, 8 had been developed involving users in the process.

Half of the apps (n=24) had their efficacy evaluated (translation apps: 2/16, 13%; health promotion apps: 22/32, 69%). Study designs were randomized controlled trials (9/24, 38%), nonrandomized trials (7/24, 29%), survey (3/24, 13%), qualitative (2/24, 8%), mixed methods (2/24, 8%), and economic analysis (1/24, 4%). The endpoints or outcomes used for those evaluations were reported behavior change (10/24, 42%), knowledge improvement (7/24, 29%), self-reported health markers, such as improved quality of life, better sleep, less anxiety (4/24, 17%), biometric health markers (2/24, 8%), cost-effectiveness (2/24, 8%), and accuracy of medical information (n=2, 8%). The total exceeds 100% as 11 apps used several different endpoints. Among the 24 apps that had their efficacy evaluated, 12 (50%) had significant positive results; 5 (12%) had partially positive results, meaning that the app showed significant efficacy in some measured outcomes but not all, or was effective in some population and not all; and 2 apps had nonsignificant results (8%); 5 studies were ongoing (21%). Details of the efficacy and acceptability studies are in Multimedia Appendix 4.

Positive outcomes (Table 3) reported for translation apps includes reducing the need to call an interpreter, especially in emergency situations [21], reduced consultation time [22,23], and reduced patient anxiety [24]. Negatives that were reported included limitations in the dialogue between health professionals and patients [25-27] and concerns about hindering the therapeutic relationship [28]. Health promotion apps had positive results in terms of acceptability and efficacy. Positive outcomes included improved quality of life and better management of chronic illnesses such as diabetes [29-31], cancer [32-34], HIV [35], depression [19,36], and addiction [37,38].

**Table 3:** Characteristics of health apps linked to better acceptability or efficacy.

App type	Important points noted during the development	Characteristics linked to better acceptability or efficacy
Translation	Many experts recommend that culturally tailored materials be created de novo or in tandem, rather than as variations on existing materials	Speech is generally preferred to text Including a button, equivalent to the patient’s “I do not understand the question” Including a phrase for health care practitioners “I don’t understand your answer” Integrating an option to directly call an interpreter in the app Integrating a list of nearby hospitals for follow-up care Including the option for patients to respond with pictures Including the option for health care practitioners to save the conversation with a patient (with respect to data protection and confidentiality)
Health promotion	Addressing both motivation as well as linguistic and sociocultural barriers and reassuring participants of confidentiality	Apps that personalize the experience for users are preferred Including a help function and a tutorial Include a tutorial provided by a virtual human rather than text Culturally appropriate, with photos of a multigenerational family Colorful and eye catching, but also professional, with easy-to-access information Easy to navigate with simple and easy-to-understand information Interactive with immediate feedback Audio, videos and pictures Provision of links for further information about a health issue Use of humor considered very effective by target audiences Including a list of frequently asked questions for users Including the option for people who have migrated to learn medical terms in the language of the country they live in

## Discussion

### Main Results

We synthesized evidence regarding the development, acceptability, and efficacy of health apps and electronic tools created to overcome the language barriers. Acceptability was evaluated in almost two-thirds of the apps and was generally high. Although health promotion or prevention programs specifically targeting people who have migrated might be complex to integrate into a health system, they are generally well accepted [39] and are a way to elevate health for all individuals.

Efficacy evaluations were only conducted for half of the apps. In those evaluations, the endpoints were mostly related to reported behavior change and knowledge improvement. Knowledge improvement, however, does not systematically lead to behavior change and the reported behavior change may not be long lasting. Changes in health outcomes are rarely measured in health promotion programs or health promotion research; this should be included as a goal of health communication tools. Indeed, a survey of researchers in health promotion highlighted that the majority of assessment measures changes in awareness, knowledge, skills, policy changes, changes in behavior, changes in community capacity, changes in organizational capacity, but changes in health outcomes are not cited [40]. In addition, several systematic reviews [41-43] examining the literature related to health promotion programs have shown that programs are rarely evaluated in terms of health outcomes. It seems, therefore, that assessments of health promotion apps are in line with current practices for assessments of health promotion programs, which focus on knowledge and reported practices but rarely on final health outcomes. Given the difficulty of measuring the health outcomes of health promotion programs or apps, it is necessary to develop new methods.

Although we could only retrieve information about the funding of 69% of apps (33/48), we found that the majority had either public or charitable funding, while only 2 apps received industry funding. Additionally, only 5 apps reported charging users a fee. That seems to suggest that both health promotion and medical translation apps mostly have a noncommercial purpose and were designed with a public health goal.

Only half of the apps had their efficacy evaluated, of which half had a significant positive result. Efficacy was evaluated more often for health promotion apps than for medical translation apps. As we have mentioned, communication difficulties between health providers and individuals with language barriers can have several negative consequences, such as less satisfaction with care [7], longer consultation time [44], lower adherence to treatment protocols [45], less health education messages delivered [9], and worse clinical outcomes [8]; therefore, it is of tremendous importance that medical translation apps are rigorously assessed, not only for their acceptability but also for their efficacy.

### Comparison With Literature

Several literature reviews [46-48] have been conducted of health apps and multimedia-based health promotion programs; however, none has specifically examined apps that are focused specifically on language barriers. Two reviewed the use of mobile health technology use and implications in historically underserved and minority populations in the United States [46] and mobile health interventions to promote physical activity for Black and Hispanic women [47] but scarcely addressed language barriers. We found one systematic review evaluating consumer health information technology interventions toward US Spanish-speaking populations [48]. The study [48] focused on one specific population in a specific country (USA), for a very wide scope of electronic interventions (eg, radio, videos, text messages services) that is different from the scope of our article. In that systematic review, the most commonly used evaluation metrics were behavior, attitude change, usability, and knowledge retention. The results of the study [48] and of our own were similar.

### Strengths and Limitations

We conducted an extensive search of the literature using 3 different databases of publications. The selection of articles was conducted by two researchers working independently; results were compared and differences were settled by a third party. Most articles were read and their data extracted by two researchers independently, and results were compared when analyzing data. This enabled us to have a higher quality of data and a reduced risk of bias. We extended the scope of our systematic review to both electronic tools designed for translation and those designed to promote health among people who have migrated with a language barrier. Although the electronic tools have seemingly different objectives, it is likely that, in the future, there will be hybrid apps that will be developed to integrate both objectives. Indeed, many primary care consultations include health education and advice from a health provider, and hospitals have been advised to include health promotion activities into their activities [49]. Development of new hybrid apps that includes both objectives will be able to learn from the evidence from the both types of apps we examined.

In this systematic review, we only included apps that were referred to in a scientific journal. Our analysis did not include the plethora of apps designed to promote heath or facilitate consultation for people who have migrated with a language barrier that were not the topic of published peer-reviewed articles. Since the objective of our review was to evaluate the evidence related to the development or evaluation of these types of apps, it was not relevant to include these other sources. In our review, we did not assess the technical characteristics of the apps studied. Indeed most articles included in this review did not give information about the technical characteristics of the apps. Many scales and evaluations systems have been created to that end [50-53]. Assessing the technical characteristics is time-consuming and not always possible as many of those apps are not available for public use. We suggest that when new apps are developed, they strive to achieve the technical qualities measured by such scales. Other systematic reviews [54] have examined an extensive range of apps designed to help communication between health care providers and people who have migrated beyond those published in peer-reviewed articles, which focus on technical characteristics, and which provide different types of information that are complementary to that provided herein.

### Implication for Policy and Conclusion

As previously mentioned, people who do not speak the language of the country have poorer access to care, longer and less satisfactory consultations, and worse clinical outcomes. In this review, we found that translation apps showed good user satisfaction but had less data on changes in the process of care (consultation length, renouncing medical care) and no data on possible changes in clinical outcomes or medicoeconomic benefits. The evaluations of health promotion apps had positive results in terms of acceptability and efficacy; however the trials on efficacy mostly used self-reported outcomes, such as self-reported behavior changes or quality of life, rather than clinical outcomes. Most trials lacked randomization or control groups, blinding, or objective measures. This lower quality makes it difficult to draw clear conclusions on efficacy.

Future apps that are developed should include evaluation of clinical and possibly medicoeconomic benefits to draw clear recommendations on their use. The apps that were the most acceptable were those that integrated features beyond simple translation, such as making appointments with health professionals on the platform or entering basic information to prepare a visit. We recommend that future translation apps are created for medical visits integrate such. In both translation and health promotion apps, including audio and video features was most appreciated by users. We recommend integrating such features in the development of new apps.

In the future, more and more health apps will be created. Given the high cost of development [55], it is essential that apps that claim to have a public health objective undergo a rigorous evaluation of their acceptability, and efficacy. Future studies should also use strong epidemiological indicators as outcomes, such as changes in health status or access to care, rather than only using reported changes in behavior and knowledge.

The aim of this systematic review was to examine the evidence related to the development, adaptation, acceptability, and effectiveness of electronic tools designed to help health care providers communicate with or promote health among people who have migrated having a low proficiency in the language of the country of origin or a low level of health literacy. Our results, especially development and characteristics associated with a better acceptability and efficacy, should be of help to public health professionals who develop new apps.

## Appendix

Multimedia Appendix 1 PRISMA reporting checklist completed.

Multimedia Appendix 2 Keywords used for the database search.

Multimedia Appendix 3 Studies quality and risk assessment.

Multimedia Appendix 4 Summary of apps studied in this systematic review.

## Abbreviations

CHEERS: Consolidated Health Economic Evaluation Reporting Standards
ICROMS: Integrated Quality Criteria for Review of Multiple Study Designs
HIV: human immunodeficiency virus
PRISMA: Preferred Reporting Items for Systematic Review and Meta-analysis
