# Supplementary material for: Electronic Tools to Bridge the Language Gap in Health Care for People Who Have Migrated: Systematic Review
Source: J Med Internet Res. 2021 May 6;23(5):e25131. doi: 10.2196/25131 (PMC8138704; doi:10.2196/25131)
Supplement: Multimedia Appendix 2 [file jmir_v23i5e25131_app2.docx]

**Appendix 2 : Keywords used for the database search**

| **Pubmed** | (Communication Barriers*[Mesh terms] OR Translating* [Mesh terms] OR Multilingualism* [Mesh terms] OR Migrant [Mesh terms] OR  Comprehension [Mesh terms] OR Emigrants and Immigrants* [Mesh terms] OR Language barrier* [Title/abstract] OR Limited English proficiency [Title/abstract] OR Multilingual [Title/abstract] OR  Communication gap [Title/abstract] OR Translation [Title/abstract] )  **AND**  (Equipment Design/instrumentation [Mesh terms] OR Nursing Informatics [Mesh terms] OR Technology* [Mesh terms] OR Multimedia* [Mesh terms] OR Audiovisual Aids* [Mesh terms] OR  Cell Phone [Mesh terms] OR Telemedicine* [Mesh terms] OR Internet/instrumentation [Mesh terms] OR Mobile applications [Mesh terms] OR Technology [Title/abstract] OR Multimedia [Title/abstract] OR  Computer-based [Title/abstract] OR Health information technology [Title/abstract] OR eHealth [Title/abstract] OR mHealth [Title/abstract] OR Telemedicine [Title/abstract] OR Mobile app* [Title/abstract]) |
| --- | --- |
|  | **Filters :**  Years : 1998- present (included)  Language : French OR English (included) |
| **Scopus** | “Communication Barriers*” OR “Translating*” OR “Comprehension” OR “Language barrier*” OR “Limited English proficiency” OR “Multilingual*” OR “Communication gap” OR “Translation” OR  “Migrant*” [Article title, abstract, keywords]  **AND**  Multimedia OR “Computer-based” OR “Health information technology” OR eHealth OR mHealth OR Telemedicine OR “Mobile app*” OR Multimedia* OR “Nursing Informatics” OR “Audiovisual Aids*” OR “Cell Phone” [Article title, abstract, keywords] |
|  | **Refine results:**  Years : 1998- present (limit to)  Document type: Article, Conference Paper, Review, Book Chapter (limit to)  Language: English, French (limit to) |
| **Embase** | 'communication barrier'/exp OR ('translating'/exp AND 'language'/exp) OR 'multilingualism'/exp OR 'comprehension'/exp OR ‘interpersonal communication'/exp OR 'language barrier*':ti,ab,kw OR 'limited english proficiency':ti,ab,kw OR 'communication gap*':ti,ab,kw  **AND**  'multimedia'/exp OR 'nursing informatics'/exp OR 'audiovisual aid*'/exp OR 'internet based intervention'/exp OR 'mobile application'/exp OR 'mobile phone'/exp OR 'telemedicine'/exp OR 'health information technology':ti,ab,kw OR ehealth:ti,ab,kw OR mhealth:ti,ab,kw OR 'mobile app*':ti,ab,kw OR 'telemedicine'/exp |
|  | **Limit to :**  Publication years : 1998-2019  Publication type : Article, Article in Press, Conference Abstract, Conference Paper, Conference Review, Review  Language: English, French |
