# Supplementary material for: Electronic Tools to Bridge the Language Gap in Health Care for People Who Have Migrated: Systematic Review
Source: J Med Internet Res. 2021 May 6;23(5):e25131. doi: 10.2196/25131 (PMC8138704; doi:10.2196/25131)
Supplement: Multimedia Appendix 3 [file jmir_v23i5e25131_app3.docx]

**Appendix 3: Studies quality and risk of bias assessment**

| **Study** | **Study type** | **Evaluation performed** | **Score section 1** | **Score section 2** | **Score section 3** | **Score section 4** | **Score section 5** | **Score section 6** | **Score section 7** | **Total score** | **Maximum score :** | **Minimum score for quality:** |
| --- | --- | --- | --- | --- | --- | --- | --- | --- | --- | --- | --- | --- |
| (1) | RCT | ICROMS (RCT) | 2 | 2 | 2 | 6 | 0 | 2 | 8 | 22 | 30 | 22 |
| (2) | RCT | ICROMS (RCT) | 2 | 3 | 3 | 3 | 2 | 2 | 8 | 23 |  |  |
| (3) | RCT | ICROMS (RCT) | 2 | 1 | 1 | 1 | 0 | 1 | 1 | 7 |  |  |
| (4) | RCT | ICROMS (RCT) | 2 | 2 | 4 | 6 | 2 | 2 | 6 | 24 |  |  |
| (5) | RCT | ICROMS (RCT) | 2 | 4 | 6 | 6 | 2 | 2 | 8 | 30 |  |  |
| (6) | RCT | ICROMS (RCT) | 2 | 2 | 3 | 6 | 2 | 2 | 8 | 25 |  |  |
| (7) | RCT | ICROMS (RCT) | 2 | 3 | 3 | 4 | 2 | 2 | 8 | 24 |  |  |
| (8) | RCT-P | ICROMS (RCT) | 2 | 4 | 3 |  |  |  | 5 | 14 | 18* | N/A |
| (9) | RCT-P | ICROMS (RCT) | 2 | 4 | 5 |  |  |  | 5 | 16 |  |  |
| (10) | RCT-P | ICROMS (RCT) | 2 | 4 | 5 |  |  |  | 6 | 17 |  |  |
| (11) | MM-P | ICROMS (NCBA) | 5 | 2 | 4 |  |  |  | 5 | 16 | 18* | N/A |
| (12) | Qual | ICROMS (QUAL) | 6 | 2 | 2 | 1 | 2 | 2 | 8 | 23 | 26 | 16 |
| (13) | Qual | ICROMS (QUAL) | 2 | 1 | 2 | 1 | 2 | 1 | 8 | 17 |  |  |
| (14) | Qual | ICROMS (QUAL) | 6 | 2 | 2 | 2 | 2 | 2 | 9 | 25 |  |  |
| (15) | Qual | ICROMS (QUAL) | 6 | 2 | 1 | 1 | 2 | 2 | 9 | 23 |  |  |
| (16) | Qual | ICROMS (QUAL) | 2 | 1 | 1 | 1 | 1 | 0 | 2 | 8 |  |  |
| (17) | Qual | ICROMS (QUAL) | 6 | 2 | 2 | 1 | 2 | 2 | 10 | 25 |  |  |
| (18) | Qual | ICROMS (QUAL) | 4 | 2 | 2 | 1 | 1 | 0 | 5 | 15 |  |  |
| (19) | Qual | ICROMS (QUAL) | 4 | 2 | 2 | 1 | 1 | 1 | 5 | 16 |  |  |
| (20) | Qual | ICROMS (QUAL) | 1 | 2 | 1 | 1 | 1 | 1 | 7 | 14 |  |  |
| (21) | Qual | ICROMS (QUAL) | 6 | 2 | 2 | 1 | 2 | 2 | 7 | 22 |  |  |
| (22) | Qual | ICROMS (QUAL) | 6 | 2 | 1 | 1 | 2 | 2 | 8 | 22 |  |  |
| (23) | Qual | ICROMS (QUAL) | 2 | 2 | 1 | 1 | 2 | 2 | 8 | 18 |  |  |
| (24) | Qual | ICROMS (QUAL) | 3 | 2 | 2 | 1 | 1 | 1 | 5 | 15 |  |  |
| (25) | Qual | ICROMS (QUAL) | 6 | 2 | 2 | 1 | 2 | 1 | 6 | 20 |  |  |
| (26) | Qual | ICROMS (QUAL) | 3 | 2 | 1 | 2 | 1 | 1 | 4 | 14 |  |  |
| (27) | Qual | ICROMS (QUAL) | 5 | 2 | 2 | 2 | 1 | 2 | 3 | 17 |  |  |
| (28) | Qual | ICROMS (QUAL) | 4 | 2 | 2 | 2 | 2 | 2 | 7 | 21 |  |  |
| (29) | Qual | ICROMS (QUAL) | 6 | 2 | 2 | 1 | 2 | 1 | 6 | 20 |  |  |
| (30) | Qual | ICROMS (QUAL) | 2 | 2 | 0 | 1 | 1 | 0 | 2 | 8 |  |  |
| (31) | Qual | ICROMS (QUAL) | 4 | 0 | 1 | 0 | 1 | 1 | 6 | 13 |  |  |
| (32) | Qual | ICROMS (QUAL) | 2 | 2 | 2 | 1 | 2 | 2 | 9 | 20 |  |  |
| (33) | Usa | ICROMS (QUAL) | 6 | 2 | 2 | 2 | 2 | 2 | 6 | 22 |  |  |
| (34) | Usa | ICROMS (QUAL) | 5 | 2 | 2 | 2 | 2 | 2 | 6 | 21 |  |  |
| (35) | Usa | ICROMS (QUAL) | 4 | 2 | 2 | 2 | 2 | 2 | 8 | 22 |  |  |
| (36) | Usa | ICROMS (QUAL) | 3 | 2 | 2 | 2 | 2 | 1 | 9 | 21 |  |  |
| (37) | Usa | ICROMS (QUAL) | 2 | 2 | 2 | 2 | 1 | 1 | 6 | 16 |  |  |
| (38) | Usa | ICROMS (QUAL) | 4 | 2 | 2 | 2 | 2 | 2 | 5 | 19 |  |  |
| (39) | Usa | ICROMS (QUAL) | 6 | 2 | 2 | 2 | 2 | 2 | 7 | 23 |  |  |
| (40) | Usa | ICROMS (QUAL) | 0 | 0 | 1 | 1 | 1 | 1 | 5 | 9 |  |  |
| (41) | Usa | ICROMS (QUAL) | 6 | 0 | 1 | 0 | 1 | 1 | 3 | 12 |  |  |
| (42) | Usa | ICROMS (QUAL) | 5 | 2 | 2 | 1 | 2 | 2 | 10 | 24 |  |  |
| (43) | Usa | ICROMS (QUAL) | 6 | 2 | 2 | 2 | 2 | 2 | 8 | 24 |  |  |
| (44) | Usa | ICROMS (QUAL) | 3 | 1 | 1 | 2 | 0 | 1 | 2 | 10 |  |  |
| (45) | Usa | ICROMS (QUAL) | 4 | 2 | 2 | 2 | 1 | 2 | 7 | 20 |  |  |
| (46) | Usa | ICROMS (QUAL) | 4 | 0 | 1 | 1 | 1 | 0 | 2 | 9 |  |  |
| (47) | Usa | ICROMS (QUAL) | 3 | 1 | 1 | 0 | 2 | 1 | 7 | 15 |  |  |
| (48) | Usa | ICROMS (QUAL) | 2 | 1 | 0 | 0 | 1 | 1 | 2 | 7 |  |  |
| (49) | Usa | ICROMS (NCBA) | 5 | 2 | 1 | 1 | 3 | 2 | 10 | 24 | 30 | 22 |
| (50) | NCBA | ICROMS (NCBA) | 4 | 2 | 4 | 2 | 3 | 2 | 10 | 27 |  |  |
| (51) | NCBA | ICROMS (NCBA) | 4 | 2 | 4 | 1 | 2 | 2 | 9 | 24 |  |  |
| (52) | NCBA | ICROMS (NCBA) | 4 | 2 | 2 | 2 | 2 | 2 | 9 | 23 |  |  |
| (53) | NCBA | ICROMS (NCBA) | 4 | 2 | 3 | 2 | 2 | 2 | 9 | 24 |  |  |
| (54) | NCBA | ICROMS (NCBA) | 2 | 1 | 4 | 1 | 2 | 2 | 9 | 21 |  |  |
| (55) | NCBA | ICROMS (NCBA) | 4 | 2 | 0 | 1 | 2 | 1 | 8 | 18 |  |  |
| (56) | NCBA | ICROMS (NCBA) | 4 | 2 | 1 | 1 | 3 | 2 | 5 | 18 |  |  |
| (57) | NCBA | ICROMS (NCBA) | 2 | 2 | 4 | 2 | 2 | 2 | 9 | 23 |  |  |
| (58) | Usa | ICROMS (NCBA) | 4 | 2 | 1 | 2 | 2 | 2 | 9 | 22 |  |  |
| (59) | NCBA | ICROMS (NCBA) | 2 | 1 | 2 | 1 | 2 | 2 | 8 | 18 |  |  |
| (60) | MEV | CHEERS |  |  |  |  |  |  |  | 27 | 48* | N/A |

* Those scores are not validated, nor part of the original ICROMS tool.

| RCT: Randomized-controlled trial  RCT-P: Protocol for a randomized-controlled trial  MM-P: Protocol for a mixed-method study  Qual: qualitative study  Usa: Usability study  NCBA: Non-controlled before-after  MEV: medico-economic evaluation | Score section 1: Clear aims and justification  Score section 2: Managing bias in sampling or between group  Score section 3: Managing bias in outcome measurements and Blinding  Score section 4: Managing bias in follow-up  Score section 5: Managing bias in other study aspects  Score section 6: Analytical rigour  Score section 7: Managing bias in reporting/ ethical considerations |
| --- | --- |

1. Dahne J., Collado A., Lejuez C.W., Risco C.M., Diaz V.A., Coles L., et al. Pilot randomized controlled trial of a Spanish-language Behavioral Activation mobile app (¡Aptívate!) for the treatment of depressive symptoms among united states Latinx adults with limited English proficiency. J Affect Disord. 2019;250:210‑7.

2. Chee W, Lee Y, Im E-O, Chee E, Tsai H-M, Nishigaki M, et al. A culturally tailored Internet cancer support group for Asian American breast cancer survivors: A randomized controlled pilot intervention study. J Telemed Telecare. juill 2017;23(6):618‑26.

3. Arora S., Burner E., Lam J., De Santos R., Menchine M. Assessing the satisfaction of mobile health (mHealth) amongst ED inner-city patients with diabetes who received the TExT-MED intervention. Acad Emerg Med. 2013;20(5):S181.

4. Bramley D., Riddell T., Whittaker R., Corbett T., Lin R.-B., Wills M., et al. Smoking cessation using mobile phone text messaging is as effective in Maori as non-Maori. N Z Med J [Internet]. 2005;118(1216). Disponible sur: http://www.embase.com/search/results?subaction=viewrecord&from=export&id=L41519518 U2 - L41519518

5. Kurth AE, Chhun N, Cleland CM, Crespo-Fierro M, Parés-Avila JA, Lizcano JA, et al. Linguistic and cultural adaptation of a computer-based counseling program (CARE+ Spanish) to support HIV treatment adherence and risk reduction for people living with HIV/AIDS: A randomized controlled trial. J Med Internet Res [Internet]. 2016;18(7). Disponible sur: https://www.scopus.com/inward/record.uri?eid=2-s2.0-84989883444&doi=10.2196%2fjmir.5830&partnerID=40&md5=411d2fff2bca090571f0ead88fb225ce

6. Thompson DA, Joshi A, Hernandez RG, Bair-Merritt MH, Arora M, Luna R, et al. Nutrition education via a touchscreen: a randomized controlled trial in Latino immigrant parents of infants and toddlers. Acad Pediatr. sept 2012;12(5):412‑9.

7. Joshi A, Amadi C, Meza J, Aguire T, Wilhelm S. Evaluation of a computer-based bilingual breastfeeding educational program on breastfeeding knowledge, self-efficacy and intent to breastfeed among rural Hispanic women. Int J Med Inf. juill 2016;91:10‑9.

8. Böge K., Karnouk C., Hahn E., Schneider F., Habel U., Banaschewski T., et al. Mental health in refugees and asylum seekers (MEHIRA): study design and methodology of a prospective multicentre randomized controlled trail investigating the effects of a stepped and collaborative care model. Eur Arch Psychiatry Clin Neurosci [Internet]. 2019; Disponible sur: http://www.embase.com/search/results?subaction=viewrecord&from=export&id=L626513542 U2 - L626513542

9. Golchert J., Roehr S., Berg F., Grochtdreis T., Hoffmann R., Jung F., et al. HELP@APP: Development and evaluation of a self-help app for traumatized Syrian refugees in Germany- A study protocol of a randomized controlled trial. BMC Psychiatry [Internet]. 2019;19(1). Disponible sur: http://www.embase.com/search/results?subaction=viewrecord&from=export&id=L627405132 U2 - L627405132

10. Henriksen L, Flaathen EM, Angelshaug J, Garnweidner-Holme L, Småstuen MC, Noll J, et al. The Safe Pregnancy study - Promoting safety behaviours in antenatal care among Norwegian, Pakistani and Somali pregnant women: A study protocol for a randomized controlled trial. BMC Public Health [Internet]. 2019;19(1). Disponible sur: https://www.scopus.com/inward/record.uri?eid=2-s2.0-85067102670&doi=10.1186%2fs12889-019-6922-y&partnerID=40&md5=ba73a54ac34d164fc1fe2515570cb47c

11. Furaijat G, Kleinert E, Simmenroth A, Müller F. Implementing a digital communication assistance tool to collect the medical history of refugee patients: DICTUM Friedland - an action-oriented mixed methods study protocol. BMC Health Serv Res. 6 févr 2019;19(1):103.

12. Avila-Garcia P., Nouri S., Cemballi A., Sarkar U., Lyles C., Aguilera A. Engaging users in the design of an mhealth, text message-based intervention to increase physical activity at a safety-net healthcare system. J Gen Intern Med. 2019;34(2):S212.

13. Buchholz SW, Sandi G, Ingram D, Welch MJ, Ocampo EV. Bilingual Text Messaging Translation: Translating Text Messages From English Into Spanish for the Text4Walking Program. JMIR Res Protoc. 2015;4(2):e51.

14. Burner E.R., Menchine M.D., Kubicek K., Robles M., Arora S. Perceptions of successful cues to action and opportunities to augment behavioral triggers in diabetes self-management: qualitative analysis of a mobile intervention for low-income Latinos with diabetes. J Med Internet Res. 2014;16(1):e25.

15. Burner E, Menchine M, Taylor E, Arora S. Gender Differences in Diabetes Self-Management: A Mixed-Methods Analysis of a Mobile Health Intervention for Inner-City Latino Patients. J Diabetes Sci Technol. 1 janv 2013;7(1):111‑8.

16. Freyne J, Pocock C, Bradford D, Harrap K, Brinkman S. Designing Technology for Assessments of CALD Patients. Stud Health Technol Inform. 2015;214:36‑42.

17. Galinato J, Montie M, Shuman C, Patak L, Titler M. Perspectives of Nurses on Patients With Limited English Proficiency and Their Call Light Use. Glob Qual Nurs Res [Internet]. août 2016;3. Disponible sur: https://www.ncbi.nlm.nih.gov/pubmed/28393085

18. Galinato J, Montie M, Patak L, Titler M. Perspectives of Nurses and Patients on Call Light Technology. Comput Inform Nurs CIN. août 2015;33(8):359‑67.

19. Goldsmith J., Young A.J., Dale L., Powell M.P. Plain Language and Health Literacy for the Oncology Family Caregiver: Examining an English/Spanish mHealth Resource. Semin Oncol Nurs. 2017;33(5):498‑506.

20. Lee K.S.K., Wilson S., Perry J., Room R., Callinan S., Assan R., et al. Developing a tablet computer-based application ('App’) to measure self-reported alcohol consumption in Indigenous Australians. BMC Med Inf Decis Mak. 2018;18(1):8.

21. Montie M, Galinato JG, Patak L, Titler M. Spanish-Speaking Limited English Proficiency Patients and Call Light Use. Hisp Health Care Int Off J Natl Assoc Hisp Nurses. 2016;14(2):65‑72.

22. Ospina-Pinillos L., Davenport T., Mendoza Diaz A., Navarro-Mancilla A., Scott E.M., Hickie I.B. Using Participatory Design Methodologies to Co-Design and Culturally Adapt the Spanish Version of the Mental Health eClinic: Qualitative Study. J Med Internet Res. 2019;21(8):e14127.

23. Padilla R, Bull S, Raghunath SG, Fernald D, Havranek EP, Steiner JF. Designing a cardiovascular disease prevention web site for Latinos: qualitative community feedback. Health Promot Pract. janv 2010;11(1):140‑7.

24. Samkange-Zeeb F, Ernst SA, Klein-Ellinghaus F, Brand T, Reeske-Behrens A, Plumbaum T, et al. Assessing the Acceptability and Usability of an Internet-Based Intelligent Health Assistant Developed for Use among Turkish Migrants: Results of a Study Conducted in Bremen, Germany. Int J Environ Res Public Health. 2015;12(12):15339‑51.

25. Silvera‐Tawil D, Pocock C, Bradford D, Donnell A, Harrap K, Freyne J, et al. CALD Assist—Nursing: Improving communication in the absence of interpreters. J Clin Nurs. 2018;27(21‑22):4168‑78.

26. Dolwick Grieb S, Flores-Miller A, Gulledge N, Clifford R, Page K. ¡Vive!: Designing an intervention to improve timely HIV diagnosis among latino immigrant men. Prog Community Health Partnersh Res Educ Action. 2016;10(3):365‑72.

27. Freyne J, Bradford D, Pocock C, Silvera-Tawil D, Harrap K, Brinkmann S. Developing Digital Facilitation of Assessments in the Absence of an Interpreter: Participatory Design and Feasibility Evaluation With Allied Health Groups. JMIR Form Res [Internet]. 9 janv 2018 [cité 5 sept 2019];2(1). Disponible sur: https://www.ncbi.nlm.nih.gov/pmc/articles/PMC6334691/

28. Ko L.K., Reuland D., Jolles M., Clay R., Pignone M. Cultural and linguistic adaptation of a multimedia colorectal cancer screening decision aid for Spanish-speaking Latinos. J Health Commun. 2014;19(2):192‑209.

29. Cameron LD, Durazo A, Ramírez AS, Corona R, Ultreras M, Piva S. Cultural and Linguistic Adaptation of a Healthy Diet Text Message Intervention for Hispanic Adults Living in the United States. J Health Commun. 2017;22(3):262‑73.

30. Rehman SA, Chen Z, Haris M. Healthcare application for foreigners living in China. 2018;590:141‑8.

31. Ruvalcaba D, Peck HN, Lyle C, Uratsu CS, Escobar PR, Grant RW. Translating/creating a culturally responsive Spanish-language mobile app for visit preparation: Case study of "trans-creation. J Med Internet Res [Internet]. 2019;21(4). Disponible sur: https://www.scopus.com/inward/record.uri?eid=2-s2.0-85067900640&doi=10.2196%2f12457&partnerID=40&md5=e0b4dcc110a0ea2b851bd7b4bce61c09

32. Jacobs RJ, Caballero J, Ownby RL, Kane MN. Development of a culturally appropriate computer-delivered tailored Internet-based health literacy intervention for Spanish-dominant Hispanics living with HIV. BMC Med Inform Decis Mak. 30 nov 2014;14:103.

33. Berry DL, Halpenny B, Bosco JLF, Bruyere Jr J, Sanda MG. Usability evaluation and adaptation of the e-health Personal Patient Profile-Prostate decision aid for Spanish-speaking Latino men eHealth/telehealth/mobile health systems. BMC Med Inform Decis Mak [Internet]. 2015;15(1). Disponible sur: https://www.scopus.com/inward/record.uri?eid=2-s2.0-84937867384&doi=10.1186%2fs12911-015-0180-4&partnerID=40&md5=54296642408779ec9c371f6d068f5834

34. Borsari L, Stancanelli G, Guarenti L, Grandi T, Leotta S, Barcellini L, et al. An Innovative Mobile Health System to Improve and Standardize Antenatal Care Among Underserved Communities: A Feasibility Study in an Italian Hosting Center for Asylum Seekers. J Immigr Minor Health. 2018;20(5):1128‑36.

35. Buscemi J, Buitrago D, Iacobelli F, Penedo F, Maciel C, Guitleman J, et al. Feasibility of a Smartphone-based pilot intervention for Hispanic breast cancer survivors: a brief report. Transl Behav Med. 7 juill 2018;

36. Day KJ, Song N. Attitudes and concerns of doctors and nurses about using a translation application for in-hospital brief interactions with Korean patients. J Innov Health Inform. 2017;24(3):916.

37. Hasegawa S., Hasegawa A., Takasu K., Kojima T., Miyao M., Sugita N., et al. Multilingual medical dialog system developed as smartphone/tablet application. Conf Proc IEEE Eng Med Biol Soc. 2013;2013:7188‑91.

38. Johnson MJ, Evans DG, Mohamed Z, Caress A-L. The development and evaluation of alternative communication strategies to facilitate interactions with Somali refugees in primary care: a preliminary study. Inform Prim Care. 2006;14(3):183‑9.

39. Joshi A, Wilhelm S, Aguirre T, Trout K, Amadi C. An Interactive, Bilingual Touch Screen Program to Promote Breastfeeding Among Hispanic Rural Women: Usability Study. JMIR Res Protoc. 2013;2(2):e47.

40. Leeman-Castillo B, Beaty B, Raghunath S, Steiner J, Bull S. LUCHAR: Using computer technology to battle heart disease among latinos. Am J Public Health. 2010;100(2):272‑5.

41. Mendu S, Boukhechba M, Gordon JR, Datta D, Molina E, Arroyo G, et al. Design of a Culturally-Informed Virtual Human for Educating Hispanic Women about Cervical Cancer. Int Conf Pervasive Comput Technol Healthc Proc Int Conf Pervasive Comput Technol Healthc. mai 2018;2018:360‑6.

42. Muroff J., Robinson W., Chassler D., López L.M., Gaitan E., Lundgren L., et al. Use of a Smartphone Recovery Tool for Latinos with Co-Occurring Alcohol and Other Drug Disorders and Mental Disorders. J Dual Diagn. 2017;13(4):280‑90.

43. Narang B., Park S.-Y., Norrmen-Smith I., Lange M., Ocampo A., Gany F., et al. The use of a mobile application to increase access to interpreters for cancer patients with limited english proficiency. J Gen Intern Med. 2018;33(2):373.

44. Oladosu JB, Emuoyibofarhe JO. A yoruba-english language translator for doctor-patient mobile chat application. Int J Comput Appl. 2012;34(3):149‑56.

45. Somers HL, Caress A-L, Evans DG, Johnson MJ, Lovel HJ, Mohamed Z. A computer-based aid for communication between patients with limited English and their clinicians, using symbols and digitised speech. Int J Med Inf. 2008;77(8):507‑17.

46. Villalobos O, Lynch S, DeBlieck C, Summers L. Utilization of a Mobile App to Assess Psychiatric Patients With Limited English Proficiency. Hisp J Behav Sci. 2017;39(3):369‑80.

47. Taicher BM, Alam RI, Berman J, Epstein RH. Design, implementation, and evaluation of a computerized system to communicate with patients with limited native language proficiency in the perioperative period. Anesth Analg. janv 2011;112(1):106‑12.

48. Im E-O, Ji X, Zhang J, Kim S, Lee Y, Chee E, et al. Issues in Developing and Evaluating a Culturally Tailored Internet Cancer Support Group. Comput Inform Nurs CIN. 2016;34(10):462‑9.

49. Paula Cupertino A, Richter K, Cox LS, Garrett S, Ramirez R, Mujica F, et al. Feasibility of a Spanish/English computerized decision aid to facilitate smoking cessation efforts in underserved communities. J Health Care Poor Underserved. 2010;21(2):504‑17.

50. Buchholz SW, Ingram D, Wilbur J, Fogg L, Sandi G, Moss A, et al. Bilingual Text4Walking Food Service Employee Intervention Pilot Study. JMIR MHealth UHealth. 2016;4(2):e68.

51. Fernández-Gutiérrez M, Bas-Sarmiento P, Poza-Méndez M. Effect of an mHealth Intervention to Improve Health Literacy in Immigrant Populations: A Quasi-experimental Study. Comput Inform Nurs CIN. 2019;37(3):142‑50.

52. Muroff J., Robinson W., Chassler D., López L.M., Lundgren L., Guauque C., et al. An Outcome Study of the CASA-CHESS Smartphone Relapse Prevention Tool for Latinx Spanish-Speakers with Substance Use Disorders. Subst Use Misuse. 2019;54(9):1438‑49.

53. Nápoles AM, Santoyo-Olsson J, Chacón L, Stewart AL, Dixit N, Ortiz C. Feasibility of a mobile phone app and telephone coaching survivorship care planning program among Spanish-speaking breast cancer survivors. J Med Internet Res [Internet]. 2019;21(7). Disponible sur: https://www.scopus.com/inward/record.uri?eid=2-s2.0-85071634655&doi=10.2196%2f13543&partnerID=40&md5=5be05ac60839f88307c0299ff76b2bee

54. Sloand E, VanGraafeiland B, Holm A, MacQueen A, Polk S. Text Message Quality Improvement Project for Influenza Vaccine in a Low-Resource Largely Latino Pediatric Population. J Healthc Qual Off Publ Natl Assoc Healthc Qual [Internet]. févr 2019; Disponible sur: https://www.ncbi.nlm.nih.gov/pubmed/30829884

55. Snipes SA, Smyth JM, Murphy D, Miranda PY, Ishino FAM. Provision increases reported PPE use for mexican immigrant farmworkers: An mhealth pilot study. J Occup Environ Med. 2015;57(12):1343‑6.

56. Solorio R, Norton-Shelpuk P, Forehand M, Montaño D, Stern J, Aguirre J, et al. Tu Amigo Pepe: Evaluation of a Multi-media Marketing Campaign that Targets Young Latino Immigrant MSM with HIV Testing Messages. AIDS Behav. 2016;20(9):1973‑88.

57. Spechbach H, Gerlach J, Mazouri Karker S, Tsourakis N, Combescure C, Bouillon P. A Speech-Enabled Fixed-Phrase Translator for Emergency Settings: Crossover Study. JMIR Med Inform. 7 mai 2019;7(2):e13167.

58. Thompson DA, Joshi A, Hernandez RG, Jennings JM, Arora M, Ellen JM. Interactive nutrition education via a touchscreen: is this technology well received by low-income Spanish-speaking parents? Technol Health Care Off J Eur Soc Eng Med. 2012;20(3):195‑203.

59. Albrecht U-V, Behrends M, Matthies HK, von Jan U. Usage of Multilingual Mobile Translation Applications in Clinical Settings. JMIR Mhealth Uhealth. 23 avr 2013;1(1):e4.

60. Gould MS, Marrocco FA, Kleinman M, Thomas JG, Mostkoff K, Cote J, et al. Evaluating iatrogenic risk of youth suicide screening programs: a randomized controlled trial. JAMA. 6 avr 2005;293(13):1635‑43.
