# Supplementary material for: Electronic Tools to Bridge the Language Gap in Health Care for People Who Have Migrated: Systematic Review
Source: J Med Internet Res. 2021 May 6;23(5):e25131. doi: 10.2196/25131 (PMC8138704; doi:10.2196/25131)
Supplement: Multimedia Appendix 4 [file jmir_v23i5e25131_app4.docx]

**Appendix 4: Summary of applications studied in this systematic review**

| **Name of application** | **Country** | **Objective of the application** | **Results of acceptability evaluation** | **Results of efficacy evaluation** |
| --- | --- | --- | --- | --- |
| DCAT (1) | Germany | To collect medical histories from non-German speaking patients and then provide physicians with a medical report | Ongoing study | Ongoing study |
| BabelDr (2) | Switzerland | To perform diagnostic interviews under emergency conditions and to reach a correct diagnosis | Overall, the doctors were satisfied with the speech interaction function and the usefulness of the system. All doctors considered that the system helped them reach a conclusion. Some doctors felt constrained by the tool and were unable to ask all the questions they wanted to. | Not evaluated |
| Visit Planner (Spanish version) (3) | USA | To help patients prepare for their primary care visits while in the waiting room. | Spanish-speaking patients spent a similar amount of time going through the Spanish version of the Visit Planner as users of the original English tool and had similar satisfaction level. | Not evaluated |
| ExLanguage (4) | Japan | To assist travelers in emergency medical situations, to support for nurses needing to speak to foreign patients and to help with communication between Japanese medical staff and foreign patients | The aim of the acceptability evaluation was to select one design, based on users preferences. | Not evaluated |
| Eloquence (5–7) | USA | To facilitate the communication around the use of call light for hospitalized patients | Good acceptability by the majority of participants | Not evaluated |
| Yoruba-English translator (8) | Nigeria | To allow Yoruba-speaking patients to reach out to physicians and have access to health care services | Users were satisfied with having tools that can assist medical practitioners in doing their job and patients in accessing medical services. | Not evaluated |
| Unnamed application  (9,10) | United Kingdom | To support communication between healthcare providers and Somali patients with limited English and literacy | Very good satisfaction. In some cases clinicians were not always sure that they had correctly understood the patient’s intended answer. Suggestions for improvement. | Not evaluated |
| PANDA (11) | Italy | To provide antenetal care to a migrant population | 90% of women considered the time of the visit to be adequate. All women were satisfied with the quality of the health education provided on nutrition, personal hygiene and family planning. | Not evaluated |
| Listen Please (12) | New Zealand | To facilitate brief dialogue between patients and health care professionals | The app is easy to use. It was considered useful for everyday brief interactions and urgent situations where there is no time to wait for an interpreter. | Not evaluated |
| CommunicatOR (13) | USA | To allow anesthesia care providers to convey information and instructions to patients and to engage in simple responsive dialogs. | Acceptance was high, with all patients indicating that they would like to use it again. 88% of patients felt more relaxed. 96% of patients understood all instructions. | Not evaluated |
| CALD Assist (14,15) | Australia | To support assessments with culturally and linguistically diverse patients when an interpreter is not present | Improvements were seen in the length of time required to complete assessments, (average time falling from 42 min to 15.6 min). The majority of participants used only positive about the app. | Not evaluated |
| Canopy (16) | USA | To improve communication between healthcare worker and migrant and reduce need for telephone interpreter | SUS score higher post test than pretest. Participants thought that a translation app might hinder the therapeutic relationship between patients and health care professionals but was more practical than other means, such as contacting translators. | Not evaluated |
| XPrompt (17) | USA | To enable the dialog between medical care staff and patients who don't share a common language. | Participants generally rated XPrompt as helpful for communicating with foreign language patients. They were less enthusiastic about xprompt’s practical use, although the app was perceived as easy-to-use. | Not evaluated |
| Unnamed- interpreter application (18) | USA | To increase the frequency of interpreter services use among providers at a comprehensive cancer center | The application increased the frequency of phone calls to the OPI service. The majority of respondents thought the app was very easy to use(82%) and helped calling interpreter with shorter waiting time to be connected. | Not evaluated |
| BUAA+ (19) | China | To help foreigners describe their medical conditions with healthcare providers | Not evaluated | Not evaluated |
| Cultural Key Phrase Tool (CKPT app) (20) | Australia | To translate clinical assessment questions in 10 common languages using pictorial, written and voice-over prompts to assist patient assessments | Not evaluated | Not evaluated |
| HELP@APP (21) | Germany | To promote self-help for Syrian refugees with posttraumatic stress symptoms | Ongoing study | Ongoing study |
| Unnamed (safe pregnancy study) (22) | Norway | To promote safety behaviour amongst pregnant women who are at risk of intimate partner violence | Ongoing study | Ongoing study |
| TExT-MED (23–25) | USA | To increase patients’ knowledge about diabetes and increase self-efficacy and disease management | The participants reported that TExT-MED helped them to take control of their diabetes and to make positive behavior choices. Specific strengths: Medication reminders messages. Weaknesses: lack of personalization. | Men had increased self-efficacy while women showed increased knowledge. Behaviour change different for men and women. |
| Interactive Bilingual Touch Screen Program (26,27) | USA | To promote breastfeeding practices among Hispanic women living in rural settings | All participants found this program easy to navigate and most found it self-explanatory. Average SUS scores 90. 60% found it very interesting, and most would use it quite often. Suggestions for improvement. | Ongoing study |
| Nuevo Amanecer (28) | USA | To help breast cancer survivors receive optimal care and manage their condition. | The majority of the women rated the overall quality of the app as very good or excellent. | Significant 2-month improvements in fatigue, health distress, and emotional well-being and increased knowledge of recommended follow-up care and average daily steps. |
| Care+ Spanish (29) | USA | To support people living with HIV in achieving medication adherence and reduce their risk of secondary HIV infections. | All usability participants reported the program was easy to use and navigate and supported privacy and confidentiality. 7/8 reported that they would prefer to use it rather than counseling with a person. On average, the tool was rated a 9/10. | Although intervention participants had reduced viral loads, increased ART adherence and decreased sexual transmission risk behaviors over time, these findings were not statistically significant |
| My Guide (30) | USA | To improve health-related quality of life amongst hispanic breast cancer survivors. | The recruitment rate was 75% and the retention rate was 91.7%; The mean satisfaction score was 65.91. Suggestions for improvement. | Scores on the knowledge significantly improved. However, there were no statistically significant changes across time. |
| Unnamed- computerized decision aid to facilitate smoking cessation (31) | USA | To facilitate the use of health resources to stop smoking. | In general, participants were highly satisfied with the app. Perceived strengths included the opportunity to discuss smoking cessation, program’s availability in Spanish, and involvement of the quitline counselor. Reported weaknesses included few follow-up calls and inadequate number of graphic images to motivate smokers. | During the decision making process, 95.3% of participants reported they were interested in using smoking cessation pharmacotherapy and 70.3% reported they were interested in using the quitline. Approximately 80% of participants set a quit date. |
| Unnamed- touchscreen educational module (32,33) | USA | To improve nutrition and feeding practices of under-3 children. | Overall, most participants reported that the touchscreen was easy or very easy to use. Regarding the content of the nutrition modules, almost all users found it very useful (95%) and very easy to understand (96%). 16%, would have preferred to receive the information on a piece of paper instead of using the touchscreen. | Improvement of knowledge on infant/toddler nutrition and feeding, especially for participants with lower educational level. 71% of participants planned to change something on the basis of what they learned from the computer program. |
| ¡Aptívate! (34,35) | USA | To deliver Brief Brief Behavioral Activation therapy to Latinx people with low english proficiency. | Nearly all participants used the app on average at least once per week and a third of participants used the app on average once per day. | Regarding efficacy, pilot trial suggests preliminary efficacy. Participants had significantly lower symptoms of depression over time. |
| CASA-CHESS (36,37) | USA | To provide 24/7 personalized emotional and instrumental support to Latin adults struggling with addiction. | 26.6% participants discontinued using CASA-CHESS, and 73.4% remained active for four or more months. Users felt connected with their CASA-CHESS case manager/counselor. | Participants who used the phone and app  significantly less likely to report use of illegal drugs at 6 months follow-up and significant reductions in depression and anxiety scores |
| Internet Cancer Support Group- (38,39) | USA | To provide emotional support, information and interactions with peers and health care professionals for cancer survivors (Asian migrants). | All users and experts positively evaluated the program, and provided their suggestions for the display, educational contents, and user-friendly structure. All of them were satisfied with the display, structure, and titles used in the program. | Significant positive changes in support care needs and symptoms of the control group from the pre-test to the post-test. Significantly greater improvements than the control group in physical and psychological symptoms and quality of life from the pre-test to the post-test |
| Text4Walk (40,41) | USA | To promote walking more amongst Hispanics. | Not evaluated | The app seems effective, but the study was performed on a population already active. Effect beyond 3 months not measured, no control group. Effective in increasing physical activity (self-reported and measured) but not health markers (BMI, waist circumference, blood pressure). |
| Grog app (42) | Australia | To help Indigenous Australians collect a detailed assessment of their drinking. | Not evaluated | Ongoing study |
| Tu amigo Pepe (43) | USA | To improve HIV testing amongst Latino MSM. | Not evaluated | Significant and immediate impact on HIV testing rates. Effective in improving attitudes, beliefs, norms and self-efficacy towards HIV testing. Improved attitudes and beliefs towards condom use but no influence on reported behavior. |
| LUCHAR (44,45) | USA | To help users increase physical activity, improve nutrition, and reduce or quit smoking. | Not evaluated | Participants showed significant improvements in fruit and vegetable consumption at the follow-up assessment and significant increases in the overall quality of diet and and physical activity levels. No impact on smoking behavior. |
| La Cocina Saludable (46) | USA | To deliver accurate, consistent, and convenient nutrition education | Not evaluated | The program is a cost-effective means to deliver food and nutrition education to large numbers of persons. |
| Unnamed- PPE text messages (47) | USA | To increase use of personal protective equipement | Not evaluated | Overall, farmworkers reported wearing more PPE at follow-up compared with baseline; however, only use of gloves and safety glasses showed significant change from baseline to follow-up. |
| Unnamed- vaccination text reminders (48) | USA | To promote influenza vaccination amongst asthmatic children | Not evaluated | Participants significantly more likely to have their children vaccinated for influenza. In Year 2, 45% of children whose families received a text message received the vaccine. |
| STOMP (49) | New Zealand | To support smoking cessation among Maori young people | Not evaluated | Intervention as effective amongst Maoris as non-Maoris. |
| Balsam (50) | Germany | To help migrants and refugees better cope with psychological distress. | Not evaluated | Ongoing study |
| e_SaludAble (51) | Spain | To improve the cognitive and social skills that allow immigrants to access and use health services | Not evaluated | Improved the cognitive and social skills required to access and use health services in participating immigrants. All indicators improved. |
| Health Communication App (52) | USA | To support the limited-English proficient cancer family caregiver. | No result of acceptability reported (only suggestions for improvement from participants) | Not evaluated |
| P3P (53) | USA | To prepare men with localized prostate cancer understand and evaluate care options, communicate priorities, and make choices. | The majority of usability issues reported were due to lack of content comprehension or translation. Participants found Spanish P3P highly acceptable. | Not evaluated |
| Intelligent Health Assistant (54) | Germany | To provide information and advice on nutrition, physical activity, overweight, diabetes and pregnancy. | Participants generally found the IHA easy to use and navigate. Bilingual information is appreciated but it is not culturally appropriate enough. | Not evaluated |
| HealthyYouTXT en Español (55) | USA | To promote healthy dietary practices. | Survey revealed strong interest in app. Participants generally perceived it as highly efficacious in promoting healthy diet and weight loss. | Not evaluated |
| Virtual Patient Educator (56) | USA | To educate Hispanic women about cervical cancer and HPV. | All participants found the VPE acceptable and attractive, most understood the material provided. Some new technical issues reported. | Not evaluated |
| MHeC-S (57) | Australia | To deliver best-practice clinical services to young people experiencing mental health problems. | There was adequate acceptability of the 5 main elements of the MHeC-S | Not evaluated |
| CHOICE (58) | USA | To increase colorectal cancer screening. | Not evaluated | Not evaluated |
| Computer-based HIV application (59) | USA | To improve medication adherence in HIV positive latinos | Not evaluated | Not evaluated |
| DIAMANTE trial (60) | USA | To increase physical activity among patients with diabetes and depression | Not evaluated | Not evaluated |
| Vive! (61) | USA | To deliver personalized information to Latino immigrant men who refuse HIV testing during outreach. | Not evaluated | Not evaluated |

**References**

1. Furaijat G, Kleinert E, Simmenroth A, Müller F. Implementing a digital communication assistance tool to collect the medical history of refugee patients: DICTUM Friedland - an action-oriented mixed methods study protocol. BMC Health Serv Res. 6 févr 2019;19(1):103.

2. Spechbach H, Gerlach J, Mazouri Karker S, Tsourakis N, Combescure C, Bouillon P. A Speech-Enabled Fixed-Phrase Translator for Emergency Settings: Crossover Study. JMIR Med Inform. 7 mai 2019;7(2):e13167.

3. Ruvalcaba D, Peck HN, Lyle C, Uratsu CS, Escobar PR, Grant RW. Translating/creating a culturally responsive Spanish-language mobile app for visit preparation: Case study of "trans-creation. Journal of Medical Internet Research [Internet]. 2019;21(4). Disponible sur: https://www.scopus.com/inward/record.uri?eid=2-s2.0-85067900640&doi=10.2196%2f12457&partnerID=40&md5=e0b4dcc110a0ea2b851bd7b4bce61c09

4. Hasegawa S., Hasegawa A., Takasu K., Kojima T., Miyao M., Sugita N., et al. Multilingual medical dialog system developed as smartphone/tablet application. Conf Proc IEEE Eng Med Biol Soc. 2013;2013:7188‑91.

5. Galinato J, Montie M, Patak L, Titler M. Perspectives of Nurses and Patients on Call Light Technology. Comput Inform Nurs. août 2015;33(8):359‑67.

6. Galinato J, Montie M, Shuman C, Patak L, Titler M. Perspectives of Nurses on Patients With Limited English Proficiency and Their Call Light Use. Global qualitative nursing research [Internet]. août 2016;3. Disponible sur: https://www.ncbi.nlm.nih.gov/pubmed/28393085

7. Montie M, Galinato JG, Patak L, Titler M. Spanish-Speaking Limited English Proficiency Patients and Call Light Use. Hisp Health Care Int. 2016;14(2):65‑72.

8. Oladosu JB, Emuoyibofarhe JO. A yoruba-english language translator for doctor-patient mobile chat application. International Journal of Computers and Applications. 2012;34(3):149‑56.

9. Johnson MJ, Evans DG, Mohamed Z, Caress A-L. The development and evaluation of alternative communication strategies to facilitate interactions with Somali refugees in primary care: a preliminary study. Inform Prim Care. 2006;14(3):183‑9.

10. Somers HL, Caress A-L, Evans DG, Johnson MJ, Lovel HJ, Mohamed Z. A computer-based aid for communication between patients with limited English and their clinicians, using symbols and digitised speech. International Journal of Medical Informatics. 2008;77(8):507‑17.

11. Borsari L, Stancanelli G, Guarenti L, Grandi T, Leotta S, Barcellini L, et al. An Innovative Mobile Health System to Improve and Standardize Antenatal Care Among Underserved Communities: A Feasibility Study in an Italian Hosting Center for Asylum Seekers. Journal of Immigrant and Minority Health. 2018;20(5):1128‑36.

12. Day KJ, Song N. Attitudes and concerns of doctors and nurses about using a translation application for in-hospital brief interactions with Korean patients. J Innov Health Inform. 2017;24(3):916.

13. Taicher BM, Alam RI, Berman J, Epstein RH. Design, implementation, and evaluation of a computerized system to communicate with patients with limited native language proficiency in the perioperative period. Anesth Analg. janv 2011;112(1):106‑12.

14. Silvera‐Tawil D, Pocock C, Bradford D, Donnell A, Harrap K, Freyne J, et al. CALD Assist—Nursing: Improving communication in the absence of interpreters. Journal of Clinical Nursing. 2018;27(21‑22):4168‑78.

15. Freyne J, Bradford D, Pocock C, Silvera-Tawil D, Harrap K, Brinkmann S. Developing Digital Facilitation of Assessments in the Absence of an Interpreter: Participatory Design and Feasibility Evaluation With Allied Health Groups. JMIR Form Res [Internet]. 9 janv 2018 [cité 5 sept 2019];2(1). Disponible sur: https://www.ncbi.nlm.nih.gov/pmc/articles/PMC6334691/

16. Villalobos O, Lynch S, DeBlieck C, Summers L. Utilization of a Mobile App to Assess Psychiatric Patients With Limited English Proficiency. Hispanic Journal of Behavioral Sciences. 2017;39(3):369‑80.

17. Albrecht U-V, Behrends M, Matthies HK, von Jan U. Usage of Multilingual Mobile Translation Applications in Clinical Settings. JMIR Mhealth Uhealth. 23 avr 2013;1(1):e4.

18. Narang B., Park S.-Y., Norrmen-Smith I., Lange M., Ocampo A., Gany F., et al. The use of a mobile application to increase access to interpreters for cancer patients with limited english proficiency. J Gen Intern Med. 2018;33(2):373.

19. Rehman SA, Chen Z, Haris M. Healthcare application for foreigners living in China. 2018;590:141‑8.

20. Freyne J, Pocock C, Bradford D, Harrap K, Brinkman S. Designing Technology for Assessments of CALD Patients. Studies in Health Technology and Informatics. 2015;214:36‑42.

21. Golchert J., Roehr S., Berg F., Grochtdreis T., Hoffmann R., Jung F., et al. HELP@APP: Development and evaluation of a self-help app for traumatized Syrian refugees in Germany- A study protocol of a randomized controlled trial. BMC Psychiatry [Internet]. 2019;19(1). Disponible sur: http://www.embase.com/search/results?subaction=viewrecord&from=export&id=L627405132 U2 - L627405132

22. Henriksen L, Flaathen EM, Angelshaug J, Garnweidner-Holme L, Småstuen MC, Noll J, et al. The Safe Pregnancy study - Promoting safety behaviours in antenatal care among Norwegian, Pakistani and Somali pregnant women: A study protocol for a randomized controlled trial. BMC Public Health [Internet]. 2019;19(1). Disponible sur: https://www.scopus.com/inward/record.uri?eid=2-s2.0-85067102670&doi=10.1186%2fs12889-019-6922-y&partnerID=40&md5=ba73a54ac34d164fc1fe2515570cb47c

23. Arora S., Burner E., Lam J., De Santos R., Menchine M. Assessing the satisfaction of mobile health (mHealth) amongst ED inner-city patients with diabetes who received the TExT-MED intervention. Acad Emerg Med. 2013;20(5):S181.

24. Burner E.R., Menchine M.D., Kubicek K., Robles M., Arora S. Perceptions of successful cues to action and opportunities to augment behavioral triggers in diabetes self-management: qualitative analysis of a mobile intervention for low-income Latinos with diabetes. J Med Internet Res. 2014;16(1):e25.

25. Burner E, Menchine M, Taylor E, Arora S. Gender Differences in Diabetes Self-Management: A Mixed-Methods Analysis of a Mobile Health Intervention for Inner-City Latino Patients. J Diabetes Sci Technol. 1 janv 2013;7(1):111‑8.

26. Joshi A, Amadi C, Meza J, Aguire T, Wilhelm S. Evaluation of a computer-based bilingual breastfeeding educational program on breastfeeding knowledge, self-efficacy and intent to breastfeed among rural Hispanic women. International journal of medical informatics. juill 2016;91:10‑9.

27. Joshi A, Wilhelm S, Aguirre T, Trout K, Amadi C. An Interactive, Bilingual Touch Screen Program to Promote Breastfeeding Among Hispanic Rural Women: Usability Study. JMIR Research Protocols. 2013;2(2):e47.

28. Nápoles AM, Santoyo-Olsson J, Chacón L, Stewart AL, Dixit N, Ortiz C. Feasibility of a mobile phone app and telephone coaching survivorship care planning program among Spanish-speaking breast cancer survivors. Journal of Medical Internet Research [Internet]. 2019;21(7). Disponible sur: https://www.scopus.com/inward/record.uri?eid=2-s2.0-85071634655&doi=10.2196%2f13543&partnerID=40&md5=5be05ac60839f88307c0299ff76b2bee

29. Kurth AE, Chhun N, Cleland CM, Crespo-Fierro M, Parés-Avila JA, Lizcano JA, et al. Linguistic and cultural adaptation of a computer-based counseling program (CARE+ Spanish) to support HIV treatment adherence and risk reduction for people living with HIV/AIDS: A randomized controlled trial. Journal of Medical Internet Research [Internet]. 2016;18(7). Disponible sur: https://www.scopus.com/inward/record.uri?eid=2-s2.0-84989883444&doi=10.2196%2fjmir.5830&partnerID=40&md5=411d2fff2bca090571f0ead88fb225ce

30. Buscemi J, Buitrago D, Iacobelli F, Penedo F, Maciel C, Guitleman J, et al. Feasibility of a Smartphone-based pilot intervention for Hispanic breast cancer survivors: a brief report. Transl Behav Med. 7 juill 2018;

31. Paula Cupertino A, Richter K, Cox LS, Garrett S, Ramirez R, Mujica F, et al. Feasibility of a Spanish/English computerized decision aid to facilitate smoking cessation efforts in underserved communities. Journal of Health Care for the Poor and Underserved. 2010;21(2):504‑17.

32. Thompson DA, Joshi A, Hernandez RG, Jennings JM, Arora M, Ellen JM. Interactive nutrition education via a touchscreen: is this technology well received by low-income Spanish-speaking parents? Technology and health care : official journal of the European Society for Engineering and Medicine. 2012;20(3):195‑203.

33. Thompson DA, Joshi A, Hernandez RG, Bair-Merritt MH, Arora M, Luna R, et al. Nutrition education via a touchscreen: a randomized controlled trial in Latino immigrant parents of infants and toddlers. Academic pediatrics. sept 2012;12(5):412‑9.

34. Dahne J., Collado A., Lejuez C.W., Risco C.M., Diaz V.A., Coles L., et al. Pilot randomized controlled trial of a Spanish-language Behavioral Activation mobile app (¡Aptívate!) for the treatment of depressive symptoms among united states Latinx adults with limited English proficiency. J Affective Disord. 2019;250:210‑7.

35. Dahne J, Collado A, Lejuez CW, Risco C, Diaz VA, Kustanowitz J, et al. ¿Aptívate!: A Spanish-language behavioral activation mobile application for delivery via primary care. Psychological Services. 2019;16(2):271‑5.

36. Muroff J., Robinson W., Chassler D., López L.M., Lundgren L., Guauque C., et al. An Outcome Study of the CASA-CHESS Smartphone Relapse Prevention Tool for Latinx Spanish-Speakers with Substance Use Disorders. Subst Use Misuse. 2019;54(9):1438‑49.

37. Muroff J., Robinson W., Chassler D., López L.M., Gaitan E., Lundgren L., et al. Use of a Smartphone Recovery Tool for Latinos with Co-Occurring Alcohol and Other Drug Disorders and Mental Disorders. J Dual Diagn. 2017;13(4):280‑90.

38. Im E-O, Ji X, Zhang J, Kim S, Lee Y, Chee E, et al. Issues in Developing and Evaluating a Culturally Tailored Internet Cancer Support Group. Comput Inform Nurs. 2016;34(10):462‑9.

39. Chee W, Lee Y, Im E-O, Chee E, Tsai H-M, Nishigaki M, et al. A culturally tailored Internet cancer support group for Asian American breast cancer survivors: A randomized controlled pilot intervention study. J Telemed Telecare. juill 2017;23(6):618‑26.

40. Buchholz SW, Ingram D, Wilbur J, Fogg L, Sandi G, Moss A, et al. Bilingual Text4Walking Food Service Employee Intervention Pilot Study. JMIR mHealth and uHealth. 2016;4(2):e68.

41. Buchholz SW, Sandi G, Ingram D, Welch MJ, Ocampo EV. Bilingual Text Messaging Translation: Translating Text Messages From English Into Spanish for the Text4Walking Program. JMIR Res Protoc. 2015;4(2):e51.

42. Lee K.S.K., Wilson S., Perry J., Room R., Callinan S., Assan R., et al. Developing a tablet computer-based application ('App’) to measure self-reported alcohol consumption in Indigenous Australians. BMC Med Inform Decis Mak. 2018;18(1):8.

43. Solorio R, Norton-Shelpuk P, Forehand M, Montaño D, Stern J, Aguirre J, et al. Tu Amigo Pepe: Evaluation of a Multi-media Marketing Campaign that Targets Young Latino Immigrant MSM with HIV Testing Messages. AIDS and Behavior. 2016;20(9):1973‑88.

44. Padilla R, Bull S, Raghunath SG, Fernald D, Havranek EP, Steiner JF. Designing a cardiovascular disease prevention web site for Latinos: qualitative community feedback. Health Promot Pract. janv 2010;11(1):140‑7.

45. Leeman-Castillo B, Beaty B, Raghunath S, Steiner J, Bull S. LUCHAR: Using computer technology to battle heart disease among latinos. American Journal of Public Health. 2010;100(2):272‑5.

46. Gould MS, Marrocco FA, Kleinman M, Thomas JG, Mostkoff K, Cote J, et al. Evaluating iatrogenic risk of youth suicide screening programs: a randomized controlled trial. JAMA. 6 avr 2005;293(13):1635‑43.

47. Snipes SA, Smyth JM, Murphy D, Miranda PY, Ishino FAM. Provision increases reported PPE use for mexican immigrant farmworkers: An mhealth pilot study. Journal of Occupational and Environmental Medicine. 2015;57(12):1343‑6.

48. Sloand E, VanGraafeiland B, Holm A, MacQueen A, Polk S. Text Message Quality Improvement Project for Influenza Vaccine in a Low-Resource Largely Latino Pediatric Population. Journal for healthcare quality : official publication of the National Association for Healthcare Quality [Internet]. févr 2019; Disponible sur: https://www.ncbi.nlm.nih.gov/pubmed/30829884

49. Bramley D., Riddell T., Whittaker R., Corbett T., Lin R.-B., Wills M., et al. Smoking cessation using mobile phone text messaging is as effective in Maori as non-Maori. New Zealand Med J [Internet]. 2005;118(1216). Disponible sur: http://www.embase.com/search/results?subaction=viewrecord&from=export&id=L41519518 U2 - L41519518

50. Böge K., Karnouk C., Hahn E., Schneider F., Habel U., Banaschewski T., et al. Mental health in refugees and asylum seekers (MEHIRA): study design and methodology of a prospective multicentre randomized controlled trail investigating the effects of a stepped and collaborative care model. Eur Arch Psychiatry Clin Neurosci [Internet]. 2019; Disponible sur: http://www.embase.com/search/results?subaction=viewrecord&from=export&id=L626513542 U2 - L626513542

51. Fernández-Gutiérrez M, Bas-Sarmiento P, Poza-Méndez M. Effect of an mHealth Intervention to Improve Health Literacy in Immigrant Populations: A Quasi-experimental Study. Comput Inform Nurs. 2019;37(3):142‑50.

52. Goldsmith J., Young A.J., Dale L., Powell M.P. Plain Language and Health Literacy for the Oncology Family Caregiver: Examining an English/Spanish mHealth Resource. Semin Oncol Nurs. 2017;33(5):498‑506.

53. Berry DL, Halpenny B, Bosco JLF, Bruyere Jr J, Sanda MG. Usability evaluation and adaptation of the e-health Personal Patient Profile-Prostate decision aid for Spanish-speaking Latino men eHealth/telehealth/mobile health systems. BMC Medical Informatics and Decision Making [Internet]. 2015;15(1). Disponible sur: https://www.scopus.com/inward/record.uri?eid=2-s2.0-84937867384&doi=10.1186%2fs12911-015-0180-4&partnerID=40&md5=54296642408779ec9c371f6d068f5834

54. Samkange-Zeeb F, Ernst SA, Klein-Ellinghaus F, Brand T, Reeske-Behrens A, Plumbaum T, et al. Assessing the Acceptability and Usability of an Internet-Based Intelligent Health Assistant Developed for Use among Turkish Migrants: Results of a Study Conducted in Bremen, Germany. Int J Environ Res Public Health. 2015;12(12):15339‑51.

55. Cameron LD, Durazo A, Ramírez AS, Corona R, Ultreras M, Piva S. Cultural and Linguistic Adaptation of a Healthy Diet Text Message Intervention for Hispanic Adults Living in the United States. Journal of Health Communication. 2017;22(3):262‑73.

56. Mendu S, Boukhechba M, Gordon JR, Datta D, Molina E, Arroyo G, et al. Design of a Culturally-Informed Virtual Human for Educating Hispanic Women about Cervical Cancer. Int Conf Pervasive Comput Technol Healthc. mai 2018;2018:360‑6.

57. Ospina-Pinillos L., Davenport T., Mendoza Diaz A., Navarro-Mancilla A., Scott E.M., Hickie I.B. Using Participatory Design Methodologies to Co-Design and Culturally Adapt the Spanish Version of the Mental Health eClinic: Qualitative Study. J Med Internet Res. 2019;21(8):e14127.

58. Ko L.K., Reuland D., Jolles M., Clay R., Pignone M. Cultural and linguistic adaptation of a multimedia colorectal cancer screening decision aid for Spanish-speaking Latinos. J Health Commun. 2014;19(2):192‑209.

59. Jacobs RJ, Caballero J, Ownby RL, Kane MN. Development of a culturally appropriate computer-delivered tailored Internet-based health literacy intervention for Spanish-dominant Hispanics living with HIV. BMC Med Inform Decis Mak. 30 nov 2014;14:103.

60. Avila-Garcia P., Nouri S., Cemballi A., Sarkar U., Lyles C., Aguilera A. Engaging users in the design of an mhealth, text message-based intervention to increase physical activity at a safety-net healthcare system. J Gen Intern Med. 2019;34(2):S212.

61. Dolwick Grieb S, Flores-Miller A, Gulledge N, Clifford R, Page K. ¡Vive!: Designing an intervention to improve timely HIV diagnosis among latino immigrant men. Progress in Community Health Partnerships: Research, Education, and Action. 2016;10(3):365‑72.
